# Anti-Tumor Effects of Sodium Meta-Arsenite in Glioblastoma Cells with Higher Akt Activities

**DOI:** 10.3390/ijms21238982

**Published:** 2020-11-26

**Authors:** Eun Jeong Lee, Jee Young Sung, Kyung Hee Koo, Jong Bae Park, Dae Hong Kim, Jaegal Shim, Chang Hoon Lee, Jongsun Park, Yong-Nyun Kim

**Affiliations:** 1 Division of Translational Science, National Cancer Center, 323 Ilsan-ro, Ilsandong-gu, Goyang-si 10408, Korea; angel3839@naver.com (E.J.L.); sungjy@ncc.re.kr (J.Y.S.); khkoo@kirams.re.kr (K.H.K.); jaegal@ncc.re.kr (J.S.); 2 Department of Cancer Biomedical Science, National Cancer Center, 323 Ilsan-ro, Ilsandong-gu, Goyang-si 10408, Korea; jbp@ncc.re.kr; 3 Division of Convergence Technology, National Cancer Center, 323 Ilsan-ro, Ilsandong-gu, Goyang-si 10408, Korea; dkim@ncc.re.kr; 4 College of Pharmacy, Dongguk University-Seoul, Goyang 10326, Korea; uatheone@dongguk.edu; 5 Department of Pharmacology, College of Medicine, Chungnam National University, Yuseong-gu, Daejeon 35015, Korea; insulin@cnu.ac.kr

**Keywords:** glioblastoma, PTEN, Akt, sodium meta-arsenite

## Abstract

Glioblastoma is a type of aggressive brain tumor that grows very fast and evades surrounding normal brain, lead to treatment failure. Glioblastomas are associated with Akt activation due to somatic alterations in PI3 kinase/Akt pathway and/or PTEN tumor suppressor. Sodium meta-arsenite, KML001 is an orally bioavailable, water-soluble, and trivalent arsenical and it shows antitumoral effects in several solid tumor cells via inhibiting oncogenic signaling, including Akt and MAPK. Here, we evaluated the effect of sodium meta-arsenite, KML001, on the growth of human glioblastoma cell lines with different PTEN expression status and Akt activation, including PTEN-deficient cells (U87-MG and U251) and PTEN-positive cells (LN229). The growth-inhibitory effect of KML001 was stronger in U87-MG and U251 cells, which exhibited higher Akt activity than LN229 cells. KML001 deactivated Akt and decreased its protein levels via proteasomal degradation in U87-MG cells. KML001 upregulated mutant PTEN levels via inhibition of its proteasomal degradation. KML001 inhibited cell growth more effectively in active Akt-overexpressing LN229 cells than in mock-expressing LN229 cells. Consistent with these results, KML001 sensitized PTEN-deficient cells more strongly to growth inhibition than it did PTEN-positive cells in prostate and breast cancer cell lines. Finally, we illustrated in vivo anti-tumor effects of KML001 using an intracranial xenograft mouse model. These results suggest that KML001 could be an effective chemotherapeutic drug for the treatment of glioblastoma cancer patients with higher Akt activity and PTEN loss.

## 1. Introduction

Glioblastomas are intra-axial tumors that originate from neuroglial cells of the central nervous system (CNS) [1,2]. Glioblastomas are known to display cellular heterogeneity with stem-like glioblastoma stem cells at the apex [3]. Glioblastoma multiforme (GBM) is the most common and aggressive malignant primary brain tumor in adults and is a major cause of morbidity and mortality among neurosurgical patients, with only 12% surviving beyond 36 months (long-term survivors) [4]. The current standard treatment strategy for glioblastoma is multimodal, involving maximal surgical resection followed by radiotherapy with concomitant and adjuvant temozolomide (TMZ) [2]. Although therapeutic advances in diagnosis have been made, glioblastoma is resistant to current treatments, such as chemotherapy and radiation, and effective treatment options are lacking. Recent insights into the biology of glioblastoma, including those from The Cancer Genome Atlas, have revealed important genetic events in human glioblastomas, such as gene amplification, mutation, and deletion [1,5]. The most frequent alterations in gliomas include dysregulation of growth factor signaling via amplification and mutational activation of receptor tyrosine kinase genes such as *EGFR*. Phosphatidylinositol-3-kinase (PI3K) signaling pathways are also activated due to genetic alteration in the phosphatase and tensin homolog (*PTEN*) tumor suppressor gene on 10q23.3 at the level of loss of heterozygosity, mutation, and methylation in at least 60% of gliomas [6].

PTEN, a tumor suppressor, is a lipid phosphatase that hydrolyses phosphate in position 3′ from phosphoinositide, thereby opposing mitogenic signaling mediated by PI3K [7]. The loss of PTEN function via its mutation or deletion leads to hyperactivation of PI3K signaling [7]. The loss of PTEN function has been linked to tumor malignancy, including metastasis and resistance to radiotherapy and chemotherapy in brain and breast cancer patients [8,9,10]. The major downstream effector of PI3K signaling is the pro-survival serine/threonine kinase, Akt [7]. Akt is activated by phosphorylation at Thr 308 (T308) by PDK1 and is fully activated by further phosphorylation at Ser 473 (S473) by mTORC2 [11]. Activated Akt phosphorylates numbers of substrates, and the majority of these substrates are involved in cell survival, proliferation, and metabolism. Uncontrolled Akt activation occurs through either overexpression and mutation, a loss of negative regulator PTEN, or the activation of PI3K pathways. These outcomes have been reported in a number of cancers including glioblastoma, breast, and prostate cancers [1,7,12]. Therefore, Akt is an attractive target for the control of cancers where the PI3K–Akt pathway is upregulated, such as glioblastoma.

Arsenic-based drugs have been used for centuries. Arsenic trioxide (As_2_O_3_), an arsenic derivative, exerts potent anti-tumor effects in vitro and in vivo [13]. As_2_O_3_ is known to be an effective inducer of apoptosis in patients with relapsed acute promyelocytic leukemia (APL) [13,14]. Because it also has a potent effect in vitro against different types of malignant leukemia as well as APL, there is increasing interest in the therapeutic development of arsenic compounds for the treatment of various solid tumors. Arsenic compounds are known to induce cell death via multiple pathways, such as reactive oxygen species (ROS) generation, JNK activation, and the activation of pro-apoptotic proteins such as Bax [13,15]. Recently, As_2_O_3_ was reported to decrease Akt protein levels in a caspase-dependent manner [16]. Akt is highly activated in glioblastomas when PTEN function is lost, so it is possible that an arsenic compound could control glioblastomas via Akt downregulation. As_2_O_3_ is poorly water-soluble and it must be dissolved with sodium hydroxide and then adjusted to physiologic pH, yielding s odium meta-arsenite (NaAsO_2_) [17]. NaAsO_2_, KML001, is a water soluble, and thus orally bioavailable arsenic compound that has been reported to have anti-tumor effects on several tumors, including prostate cancer and glioma cells [17,18]. KML001 is known to exerts its cytotoxic effects via its binding to telomeric sequences, which leads to shortened telomeres in prostate cancer [17,18]. KML001 has anti-tumoral effects on non-Hodgkin lymphoma cells via inhibiting cell signaling including PI3K/Akt and MAPK [13,14]. Recently, KML001 exhibits anti-tumoral effects in multiple myeloma cells via Akt inactivation and PTEN activation [19]. In addition, KML001 has entered Phase I/II clinical trials for treatment of prostate cancer [20] and Phase I clinical trials for advanced non-small-cell lung cancer as well as other platinum-responsive malignancies (https://clinicaltrials.gov). Because KML001 induces Akt inactivation and PTEN activation [19] and because Akt activation with PTEN mutation is associated with glioblastoma, in this study, we evaluated the anti-tumor effects of KML001 using PTEN-negative human glioblastoma U87-MG cells in vitro and in vivo, and investigated the possible mechanisms involved in this process. 

## 2. Results 

### 2.1. Expression Levels of PTEN and Akt Activation in Human Glioma Cell Lines

**Figure 1:**
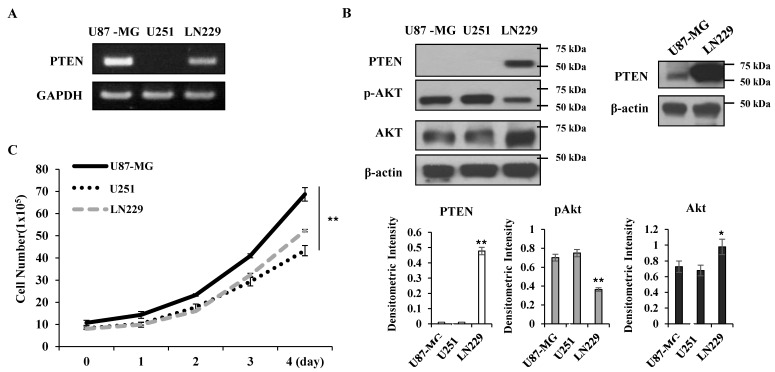
Expression levels of PTEN and Akt and cell growth in human glioblastoma cell lines. (**A**) Total RNA was extracted from glioma cell lines as indicated and subjected to RT-PCR to measure PTEN mRNA levels. (**B**) Cells were lysed and 30 μg of total cellular proteins was analyzed by immunoblotting using anti-PTEN, -pAkt, -Akt, and β-actin antibodies. β-actin was used as a loading control. In the right panel, film was exposed a little longer for PTEN immunoblot. The levels of proteins were quantified by a densitometry and normalized to the loading control β-actin (lower panel). (**C**) 1 × 10^5^ cells were seeded and were grown in the culture media condition for 4 days. The cell numbers of each cell line were counted after trypan blue-staining at indicated times. Statistical analysis was conducted using parametric one-way ANOVA test and post hoc test (Bonferroni correction). Error bars represent standard deviations of the mean of three measurements (* *p* < 0.05, ** *p* < 0.01). These experiments were performed three independent times with comparable results.

First, we examined the level of PTEN expression and Akt activation in several glioma cell lines, including U87-MG, LN229 and U251 cells. As shown in Figure 1A, when PTEN mRNA levels were evaluated by RT-PCR, PTEN expression was undetectable in U251 cells, but both U87-MG and LN229 cells expressed comparable levels of PTEN mRNA. However, protein levels of PTEN were only detected in LN229 cells (Figure 1B). U87-MG cells are known to contain in-frame deletion of Exon 3 within the tensin region [21]. Although comparable levels of this mutation were expressed at the transcriptional level, the mutant PTEN protein was not detected by immunoblotting in U87-MG cells (Figure 1B left panel and lower panel). Akt activation was higher in U87-MG and U251 cells with little PTEN expression, as assessed by immunoblotting using anti-phospho-Akt (pAkt), although Akt expression was higher in LN229 cells (Figure 1B). However, longer exposure of immunoblot could recapitulate mutant PTEN protein in U87 cells (Figure 1B, right panel). This mutation may be critical for PTEN turnover, or the antibody might not recognize this mutant protein in the same way as it does the wild-type PTEN. When cell growth rates were compared, U87-MG cells grew faster than U251 and LN229 cells (Figure 1C). These data indicate that PTEN downregulation was positively correlated with elevated Akt activation in the glioma cell lines tested in this study. 

### 2.2. Effects of KML001 on Glioma Cell Growth and Akt Activity

**Figure 2:**
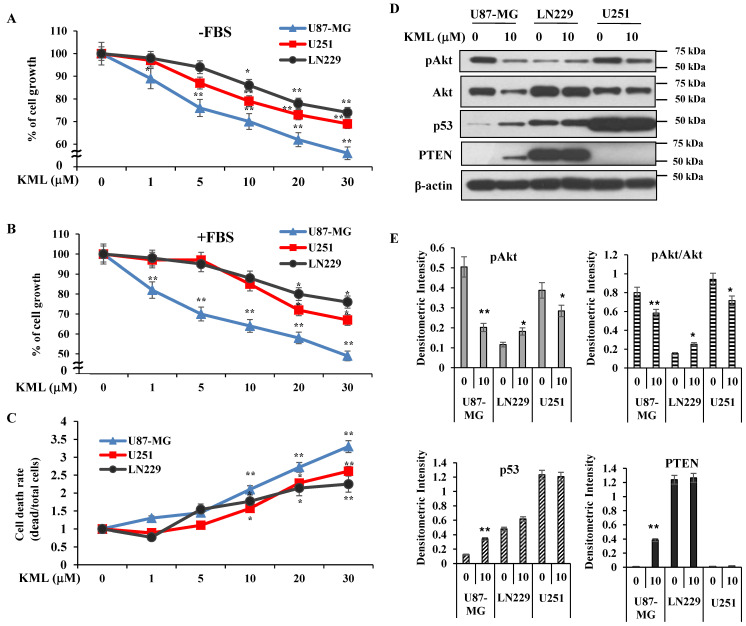
Effects of KML001 on cell growth in human glioblastoma cell lines. (**A**,**B**) Cells were treated with 0–30 μM KML001 in the culture media without or with FBS for 24 h. Cell growth was measured by MTS assay as described in Section 4. (**C**) Cells were treated with 0–30 μM KML001 in the culture media for 24 h and cells were collected, mix an equal volume of trypan blue dye and manually counted by countess^TM^ automated cell counter. (**D**) Cells were treated with 10 μM KML001 for 24 h and cell lysates were analyzed by immunoblotting using indicated antibodies. (**E**) The levels of proteins were quantified by a densitometry and normalized to β-actin. Data are presented as mean ± SE of three independent experiments (* *p* < 0.01, ** *p* < 0.05). These experiments were performed three independent times with comparable results.

To test whether KML001 could induce cell growth inhibition in glioma cells, glioma cells were treated with various concentrations of KML001 for 24 h in either serum-free medium or 10% FBS-containing medium. Treatment of KML001 resulted in a dose-dependent cell growth inhibition, regardless of serum presence, in all the cell lines tested in our study (Figure 2A,B, and Supplementary Figure S1). Compared with other cell lines, U87-MG cells, which express a mutant PTEN and possess higher Akt activity, showed higher sensitivity to lower doses of KML001. U251 cells are PTEN-negative and were also notably responsive to KML001. LN229 cells were the least responsive to KML001. To test whether KML001-induced growth inhibition was due to cell death, we carried out a trypan blue dye exclusion assay after KML001 treatment. Cell death increased in a dose-dependent manner in all three cell lines, and U87-MG cells showed greater cell death after KML001 treatment than the other two cell lines (Figure 2C). Akt activation is frequent in glioma because PI3K–Akt pathways are often activated by either growth factor receptor signaling or loss of function of PTEN [6,22]. Activated Akt exerts its pro-survival activity by phosphorylating various proteins for survival signaling. As the cell lines we used expressed different levels of pAkt, we tested whether KML001 affected Akt activity. Because 10 μM KML001 inhibited cell growth of U87-MG cells significantly when compared with other cell lines, we used 10 μM KML001 to examine changes of Akt activity in three cell lines. Upon 10 μM KML001 treatment, Akt phosphorylation and Akt protein levels decreased in U87-MG and U251 cells, whereas both pAkt and Akt protein levels increased in LN229 cells in same dose of KML001, which were the least responsive to KML001 (Figure 2D,E). Interestingly, there were detectable levels of p53 expressed in LN229 and U251 cells initially but there was little p53 expression in U87-MG cells. However, p53 was dramatically upregulated following KML001 treatment in the U87-MG cells but not in U251 or LN229 cells (Figure 2D,E). U87-MG cells are known to be PTEN-deficient [23] and we could not detect PTEN at the protein level, although its mRNA levels were notable (Figure 1A,B). However, KML001 increased the PTEN level in U87-MG cells, which exhibited non-detectable levels of PTEN initially (Figure 2D). These findings indicate that KML001 induced growth inhibition depending on Akt activity and PTEN expression status. 

### 2.3. KML001 Induced Apoptosis in U87-MG Cells

**Figure 3:**
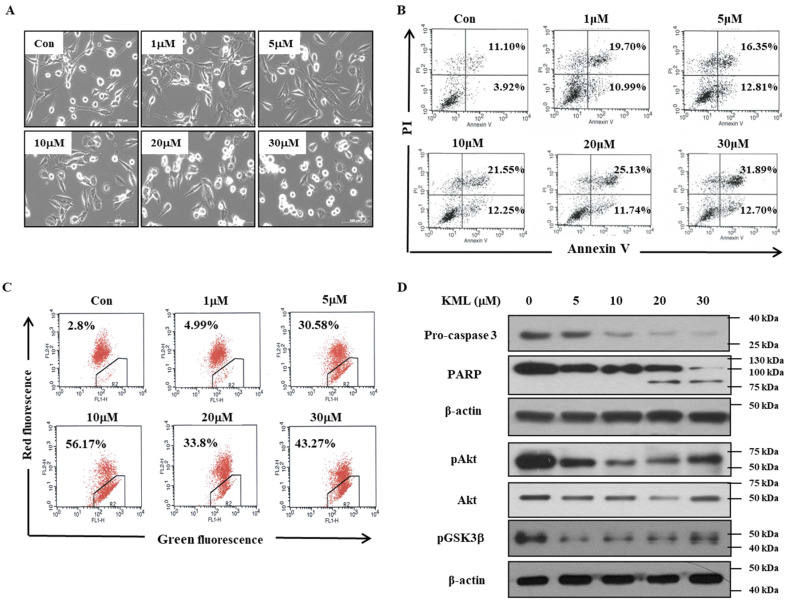
Induction of apoptosis with KML001 in U87-MG cells. (**A**) U87-MG cells were treated with 0–30 μM KML001 for 24 h and cell morphology was observed using an optical microscope. Scale bar, 100 μm. (**B**) U87-MG cells were treated with 0–30 μM KML001 for 24 h, stained with annexin V-FITC and PI, and subjected to flow cytometry analysis. Fluorescence dot blots of annexin V-positive (horizontal axis) and PI-positive (vertical axis) cells are shown. Cells that were positively stained by annexin V-FITC only (early apoptosis) and positive for both annexin V-FITC and PI (late apoptosis) were quantitated, and both subpopulations were considered as overall apoptotic cells. (**C**) U87-MG cells were treated with 0–30 μM KML001 for 24 h, and then ΔΨ_m_ was measured using the JC-1 kit by flow cytometer. The loss of ΔΨ_m_ was equated with decreased red fluorescence. (**D**) U87-MG cells were treated with 0–30 μM KML001 for 24 h and cell lysates were subjected to immunoblotting analysis using the indicated antibodies. The levels of proteins were quantified by a densitometry and normalized to β-actin (Supplementary Figure S2) Similar results were observed in three independent experiments.

To test whether the KML001-induced cell growth inhibition was associated with apoptosis, we first examined cell morphological changes. KML001 treatment induced cell rounding, which is associated with loss of adhesion (Figure 3A). Next, KML001-treated cells were stained with annexin-V-FITC and PI and processed for flow cytometry analysis. There was an increase in the annexin-V-positive population in KML001-treated cells, indicating that KML001 induced apoptosis of U87-MG cells (Figure 3B). To evaluate the involvement of the mitochondria pathway in KML001-induced cell death, we examined mitochondrial membrane potential changes using a JC-1 fluorescent probe. JC-1 selectively enters mitochondria and reacts to changes in the ratio of green and red fluorescence, indicating changes of mitochondrial membrane potential [24]. There was a decrease in red fluorescence and an increase in green fluorescence after KML001 treatment (Figure 3C), suggesting a dose-dependent decrease in mitochondrial membrane potential. As caspase-3 is also activated by the mitochondrial pathway, we investigated whether caspase-3 was activated. There was a decrease in caspase-3 pro-form and an increase in cleavage of PARP, which is a substrate for activated caspase-3. Consistent with the results in Figure 2D, KML001 reduced Akt activity in a dose-dependent manner, and consequently decreased phosphorylation of GSK3β, which is a substrate of Akt [25] (Figure 3D). These data indicate that KML001 induced apoptosis of U87-MG cells and that activation of the mitochondrial pathway and inactivation of Akt pathways are involved in this process.

### 2.4. KML001 Regulated Protein Levels of Akt and PTEN in U87-MG Cells

**Figure 4:**
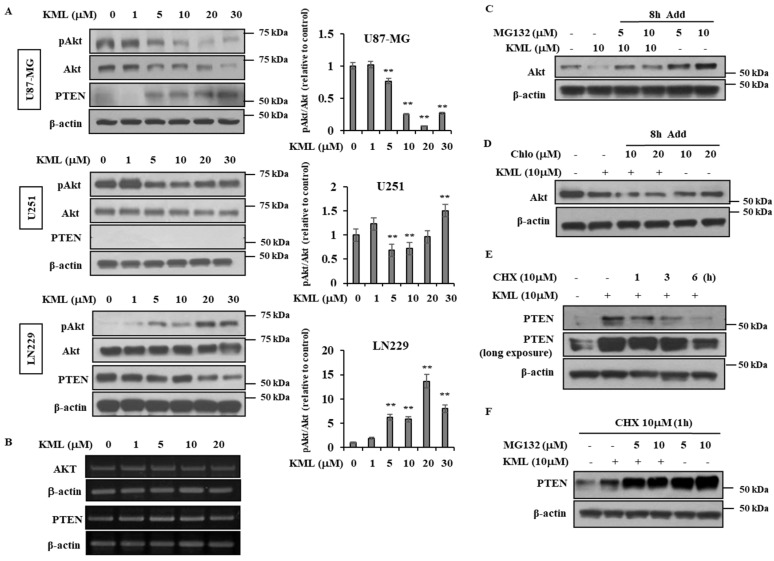
KML001-induced Akt downregulation and PTEN upregulation in U87-MG cells. (**A**) Cells were treated with 0–30 μM KML001 for 24 h and cell lysates were subjected to immunoblotting analysis using indicated antibodies. The histogram shows the densitometric measuring of pAkt/Akt ratio relative to control. pAkt/Akt ratio = [(pAkt/β-actin)/(Akt/β-actin)] (right panel). Data are presented as mean ± SE of three independent experiments. ** *p* < 0.01 indicate significant difference from the control. (**B**) U87-MG cells were treated with 0–20 μM KML001 for 24 h and total RNA was extracted and subjected to RT-PCR to assess Akt and PTEN mRNA levels. The levels of RNA were normalized with β-actin. (**C**–**E**) U87-MG cells were treated without or with 10 μM KML001 for 24 h and some KML001-treated cells were incubated with 5 μM or 10 μM MG132 (**C**), chloroquine (Chlo) (**D**), and 10 μM cycloheximide (CHX) (**E**) for indicated times before harvest. Cell lysates were subjected to immunoblotting analysis using indicated antibodies. (**F**) KML001-treated U87-MG cells were incubated with MG132 for 8 h and with 10 μM cycloheximide for 1 h before harvest. The levels of proteins were quantified by a densitometry and normalized to β-actin (Supplementary Figure S5) Cell lysates were subjected to immunoblotting analysis using indicated antibodies. These experiments were performed three independent times with comparable results.

U87-MG cells are the most responsive to KML001, so we used them to further investigate a possible mechanism by which KML001 might induce apoptosis. KML001 caused a greater decrease in levels of pAkt and total Akt in U87-MG and U251 cells than in LN229 cells (Figure 4A). The ratio of pAkt/Akt was also decreased by the KML001. In addition, PTEN protein levels increased in U87-MG cells but decreased in LN229 cells (Figure 4A). Heat shock protein 90 (Hsp90), a molecular chaperone, binds to and inhibits proteosomal degradation of its oncogenic client proteins, including soluble kinases (e.g., Akt, bcr-abl, NPN-ALK, Raf-1, and v-Src) [26]. As Akt protein levels decreased in the KML001-treated U87 cells, we examined whether or not Hsp90 protein was downregulated. There was little change in HSP90 levels, as seen in Supplementary Figure S3. When mRNA levels of Akt and PTEN were evaluated by RT-PCR their expression levels remained unchanged in the KML001-treated U87-MG cells (Figure 4B). To further investigate how Akt protein is downregulated, we used inhibitors for proteasomal degradation pathways—MG132 and lactacystin—and found that they could attenuate KML001-induced Akt downregulation, indicating that proteasomal degradation appeared to be responsible for Akt downregulation (Figure 4C, Supplementary Figure S4). However, chloroquine (a lysosomal inhibitor) did not block KML001-induced Akt downregulation (Figure 4D). In Figure 4A, PTEN protein levels were elevated in the KML001-treated U87-MG cells. KML001-induced PTEN upregulation was attenuated by cycloheximide, a protein-synthesis inhibitor (Figure 4E). MG132, a proteasomal inhibitor, accumulated PTEN dramatically even in the absence of KML001 (Figure 4F) indicating that although this mutant PTEN protein was synthesized, it was not stabilized and thus degraded. It is probable that Exon 3 of PTEN is critical for PTEN protein stability, although this remains to be elucidated. 

**Figure 5:**
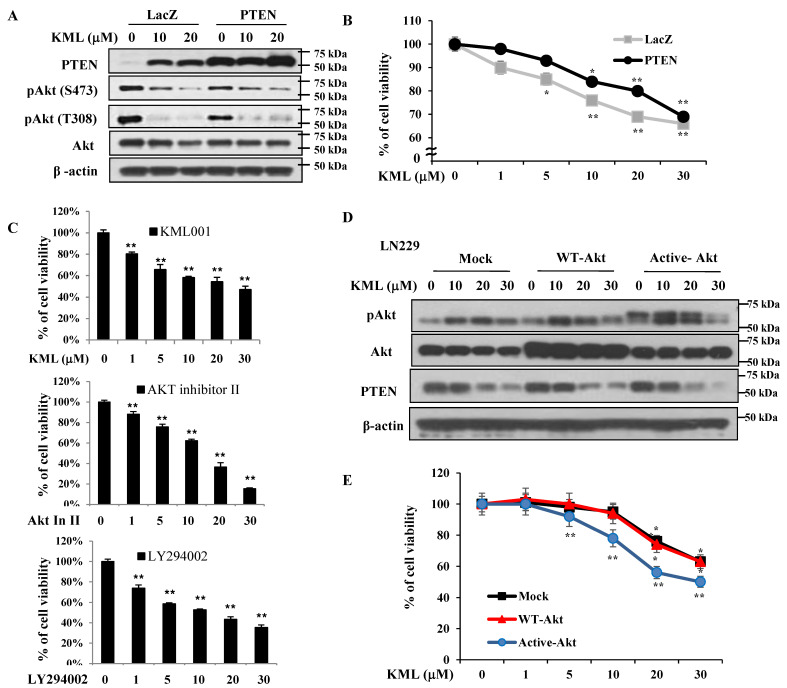
KML001-induced cell growth inhibition is correlated with Akt activity in glioblastoma cell lines. (**A**,**B**) U87-MG cells were transfected with lentivirus either control virus only (LacZ) or PTEN and cells were treated with 0–30 μM KML001 for 24 h followed by immunoblotting analysis using indicated antibodies (**A**), the levels of proteins were quantified by a densitometry and normalized to β-actin (Supplementary Figure S6) and MTS assay (**B**). (**C**) U87-MG cells were treated either with 0–30 μM KML001, 0–50 μM Akt inhibitor II, or 0–100 μM LY294002 (PI3K inhibitor) and cell growth was measured by MTS assay. (**D**,**E**) LN229 cells were infected with lentivirus encoding either vector only (Mock), wild type Akt (WT-Akt), or constitutively-active Akt (Active-Akt) and stable cell lines were established as described in the “Materials and Methods”. Cells were treated with 0–30 μM KML001 for 24 h followed by immunoblotting analysis using indicated antibodies (**D**), the levels of proteins were quantified by a densitometry and normalized to β-actin (Supplementary Figure S6) and MTS assay (**E**). Error bars represent standard deviations. * *p* < 0.05, ** *p* < 0.01 versus KML001-treated Mock-LN229 cells. These experiments were performed three independent times with comparable results.

To investigate the association of KML001 sensitivity with PTEN and Akt activity, we overexpressed wild-type PTEN in U87-MG cells. Upon PTEN overexpression, levels of Akt full activation as determined by its dual phosphorylation (S473 and T308) [11] decreased initially (Figure 5A), and sensitivity to KML001 also decreased compared to mock cells (Figure 5B). We compared the effects of KML001 on U87-MG cell growth inhibition with that of other reagents, such as Akt inhibitor II and PI3K inhibitor LY294002. All three reagents induced comparable levels of cell growth inhibition (Figure 5C), suggesting that Akt inactivation is effective for U87-MG growth inhibition. To further determine whether Akt activity is critical for KML001-induced cell death, LN229 cells were infected with lentiviral vectors expressing either Mock, wild-type (WT-Akt) or dominant-active Akt (active-Akt). Akt protein levels and Akt activity from each cell line were verified by immunoblotting using anti-pAkt and anti-Akt antibodies, respectively, as shown in Figure 5D. Basal Akt activity was higher in the active-Akt-expressing LN229 cells. WT-Akt cells showed a little more Akt activity, although Akt levels were higher than in any other cell lines (Figure 5D). It is probable that LN229 cells express high levels of WT-PTEN, which are enough to control Akt activity. Up to 20 μM KML001 treatment appeared to induce Akt activation in all three LN229 cells expressing either Mock, WT-Akt, or Active-Akt with decreased PTEN levels but 30 μM KML001 treatment decreased Akt activation even with a decreased PTEN. In addition, KML001-induced growth inhibition was prominent in the active-Akt expressing cells when compared with Mock and WT-Akt cells (Figure 5E). These data indicate that KML001 induces cell growth inhibition more sensitively in the cells with higher Akt activity.

### 2.5. Sensitivity of KML001 to Akt and PTEN Status in Other Cancer Types

**Figure 6:**
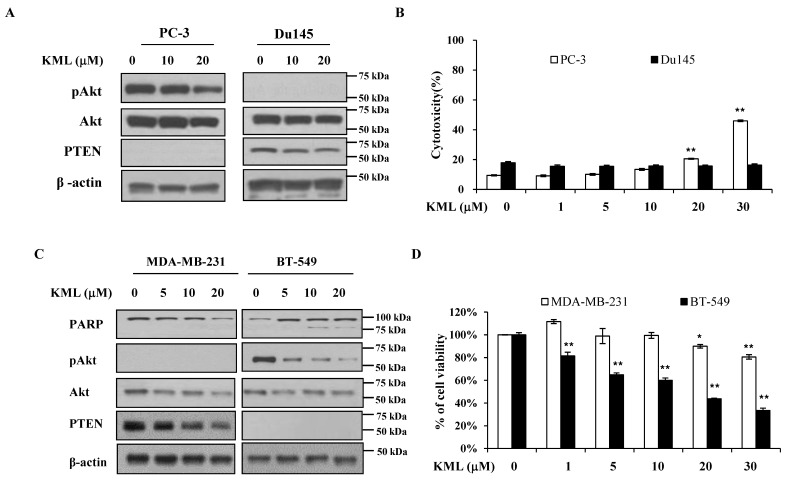
Association of KML001-induced cell growth inhibition with Akt activity and PTEN expression. (**A**,**B**) PC-3 and Du145 cells were treated with 0–30 μM KML001 for 24 h, followed by immunoblotting analysis using the indicated antibodies (**A**), the levels of proteins were quantified by a densitometry and normalized to β-actin (Supplementary Figure S7) and LDH release assay for cytotoxicity (**B**). (**C**,**D**) MDA-MB231 and BT-549, human breast cancer cells were treated with 0–30 μM KML001 for 24 h. Cells were processed for immunoblotting analysis using the indicated antibodies (**C**), The levels of proteins were quantified by a densitometry and normalized to β-actin (Supplementary Figure S7) or cell viability assay by counting of trypan blue-stained cells using countess^TM^ automated cell counter (**D**). Error bars represent standard deviations (* *p* < 0.05, ** *p* < 0.01). Similar results were observed in three independent experiments.

To test whether KML001’s effects on Akt and PTEN are limited to glioma cell lines, we employed prostate (Du145 and PC-3) and breast cancer (MDA-MB-231 and BT-549) cell lines with different PTEN expression statuses. DU145 cells are PTEN-positive and showed low levels of Akt activation (pAkt), whereas PC-3 cells are PTEN-negative and exhibited high levels of Akt activation (Figure 6A). KML001 treatment decreased Akt activation and increased cell death in PC-3 cells (Figure 6B). Similarly, MDA-MB-231 cells with PTEN exhibited little Akt activation, whereas PTEN-negative BT-549 cells showed Akt activation (Figure 6C). KML001 induced cell growth inhibition to a greater extent in the BT-549 cells than in the MDA-MB-231 cells with an increased PARP cleavage (Figure 6C,D). These data indicate that KML001-induced cell growth inhibition and/or cell death was not limited to Gio cell lines and that the effects of KML001 were greater in cells with higher Akt activation due to the absence of PTEN. 

### 2.6. Anti-Tumor Effects of KML001 in U87-MG Orthotopic Xenograft Models

**Figure 7:**
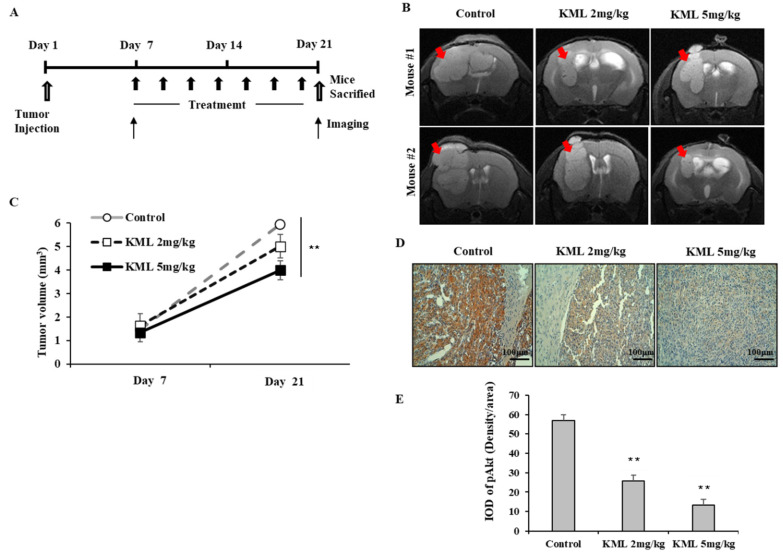
Anti-tumor effects of KML001 in orthotropic xenograft models. (**A**) Schematic procedure of in vivo experiments. On Day 1, U87-MG cells were injected into the mouse brain as described in the “Materials and Methods”. On day 7, the mice (n = 5/group) were treated with KML001 or with PBS (control) orally with 2-day intervals for two weeks and on day 21 the mice were sacrificed. On day 7 and day 21 MRI images of brains were captured. Similar results were observed in two independent experiments. (**B**) Brain MRI images of mice were captures on day 21. Representative pictures indicate mouse brain regions of corpus clausum and its surrounding structures. (**C**) Tumor volumes were measured as described in the “Materials and Methods”. Statistical analysis was conducted using one-way ANOVA test and post hoc test (Bonferroni correction), ** *p* < 0.01. (**D**) Tumor tissues were fixed and stained with anti-pAkt antibodies. Scale bars = 100 μm. White arrows indicate tumors. (**E**) The integrated optical density (IOD) was analyzed for pAkt quantification per histological field (×200 magnification). Error bars represent standard deviations (** *p* < 0.05 vs. control). Similar results were observed in three independent experiments.

To test tumor growth inhibition by KML001, we employed U87-MG orthotopic xenograft models by injecting U87-MG cells stereotactically into the left caudate-putamen region of mice. As illustrated in Figure 7A, seven days after tumor implantation, the mice were treated either with 2 mg/kg or 5 mg/kg of KML001; according to xenograft models, these treatments exert low toxicity when used in vivo over two weeks [18]. Brain tumor images were captured on day 7 and day 21 (Figure 7B) and tumor volumes were measured (Figure 7C). KML001 reduced tumor growth, and this anti-tumor effect was more obvious in the 5 mg/kg treatment (Figure 7C). When tumor tissues were analyzed by immunohistochemistry using anti-pAkt antibodies, Akt activation was reduced in the KML001-treated specimen. These data suggest that KML001 had an anti-tumor effect on glioma cells in vitro and in vivo via Akt inactivation.

## 3. Discussion

We evaluated the effects of KML001 on the growth inhibition of glioma cells, which express different levels of active-Akt depending on expression levels and/or status of PTEN, which is a negative regulator for Akt activation. We demonstrated that KML001 exhibited anti-tumor effects via the induction of glioma cell death, with a greater effect in cells with higher Akt activation. KML001 exerts its growth inhibition through Akt downregulation via the proteasomal degradation pathway. KML001 also reduced tumor growth in an orthotopic xenograft mouse model. 

Glioblastoma is the most common malignant type of primary brain tumor and is classified as a Grade IV astrocytoma by the World Health Organization, with a median survival of approximately 15 months following intensive therapy including surgery, chemotherapy, and radiotherapy [27]. The standard therapy options available to patients are minimally effective; thus, development of a novel treatment regimen is necessary to improve the survival rate among glioblastoma patients. A number of genetic and epigenetic alterations have been identified in glioblastoma that lead to the dysregulation of signaling pathways, including activation of the receptor tyrosine kinase pathway, PTEN–PI3K–Akt activation, and inhibition of p53 [28]. Therefore, drugs targeting these commonly observed alterations have been investigated as potential targeted therapies for glioblastoma [28]. The PI3K–Akt signaling pathway is activated in most glioblastoma via either growth factor receptor activation, such as EGFR, or through loss of PTEN, a negative regulator of Akt activation [28]. Therefore, Akt inhibitors may be beneficial for control of glioblastoma growth. In our study, we showed that sodium meta-arsenite (KML001, NaAsO_2_) inhibited Akt activation via its downregulation and induced apoptosis of glioblastoma cells.

Arsenic has been used to treat diseases for centuries. Among the various arsenicals, arsenic trioxide (ATO, As_2_O_3_), an FDA-approved drug, is a highly effective drug for treating acute promyelocytic leukemia (APL) with low toxicity [29]. ATO induces apoptosis of various human solid tumor cell lines, including lung cancer, breast cancer, and glioblastoma cells [15,18,30,31]. ATO is known to exert its anti-tumor effects via multiple pathways, including the mitochondrial aggregation pathway, autophagic cell death pathway, and inhibition of telomerase activity and Notch pathway [18,32,33,34]. ATO has been administered via intravenous injection for APL treatment. KML001, an ATO derivative, is water-soluble and orally bioavailable, and expresses cytotoxic activity in various cancer cells, including prostate cancer and AML [17,35]. KML001 has been demonstrated to bind to telomeres and to cause telomerase erosion in prostate cancer cells [17]. In addition, KML001 inhibits the proliferation of non-Hodgkin’s lymphoma (NHL) cell lines, while ATO is not effective. The anti-tumor effect of KML001 in NHL is mediated via the inhibition of cell signaling pathways, including STAT, PI3K–Akt, MAPK, and NF-kB signaling [14]. It has been reported that KML001 makes glioblastoma cells more sensitive to temozolomide chemotherapy and radiotherapy by enhancing DNA damage [18]. However, the anti-tumor effects of KML001 and the mechanism of this effect as related to glioblastoma have not been investigated. In this study, we found that KML001 induced cell growth inhibition to a greater extent in PTEN-negative glioblastoma cells than in PTEN-positive cells. 

PTEN is a negative regulator for Akt [12]; thus, Akt activity was higher in the PTEN-negative U87-MG and U251 cells than the PTEN-positive LN229 cells (Figure 1A,B). KML001 induced growth inhibition in glioma cells, but the degree of inhibition was more prominent in the U87-MG and U251 cells. When PTEN was overexpressed in U87-MG cells, KML001-induced cell death was attenuated (Figure 5A,B). PTEN-dependent KML001 sensitivity appears not to be limited to glioma cells, as KML001 sensitized cell growth inhibition in other PTEN-negative cells (PC-3 and BT549) when compared with PTEN-positive cells (DU145 and MDA-MB-231) (Figure 6). In addition, when WT-Akt or active-Akt were overexpressed, KML001 induced cell death to a greater extent in the active-Akt-expressing cells than in WT-Akt-expressing cells (Figure 5D,E). These data suggest that KML001 might efficiently target cancers with Akt activation either due to PTEN deletion or PI3K activation. 

KML001 treatment decreased protein levels of Akt with little change in its mRNA levels (Figure 4). Proteasomal inhibitors, namely MG132 and lactacyctin, attenuated KML001-induced Akt downregulation, but a lysosomal inhibitor, chloroquine, did not (Figure 4C,D, Supplementary Figure S4), indicating that KML001 negatively regulates Akt via its proteasomal degradation. HSP90 is a molecular chaperon for oncogenic proteins such as EGFR, Akt, and Src [36]. Although levels of HSP90 were not changed (Supplementary Figure S3), we cannot rule out the possibility that HSP90 inactivation is involved in Akt downregulation, because KML001 is known to deactivate HSP90 [37]. U87-MG cells expressed a mutant form of PTEN [23], which was expressed at the mRNA level, but hardly detected at the protein level (Figure 1). However, KML001 increased the amount of mutant PTEN at the protein level in U87-MG cells, whereas it decreased WT-PTEN levels in LN229 cells (Figure 4A). This mutant PTEN is stabilized by MG132, a proteasomal inhibitor, even without KML001 treatment (Figure 4F), indicating that Exon 3 of PTEN might be involved in its stability. 

PTEN and p53 are very well-known tumor suppressors and their dysregulations are often associated with tumor progression [38]. PTEN is known to regulate p53 protein levels and activities through both its phosphatase-dependent and phosphatase-independent mechanisms. The onset of tumor development in *p53*^−/−^*PTEN*^−/−^ mice is similar to that of *p53*^−/−^ animals, and p53 protein levels are dramatically reduced in *PTEN*^−/−^ cells and tissues. Reintroducing wild-type or phosphatase-dead PTEN mutants leads to a significant increase in p53 stability [38]. PTEN-deficient U87-MG cells possess WT-p53 but express very low levels of p53. PTEN-deficient U251 cells expressed high levels of mutant p53 initially and regardless of KML001 treatment (Figure 2D). KML001 treatment led to increases in both mutant PTEN (Figure 4) and p53 levels (Figure 2D and Supplementary Figure S8). KML001 also increased phosphorylation of p53 at Ser-46 (Supplementary Figure S8A), which is mainly involved in apoptosis [39], and induced p53 localization in the nucleus (Supplementary Figure S8B). Accordingly, p53-responsible pro-apoptotic proteins such as PUMA and Bax [40] were upregulated by KML001 (Supplementary Figure S8A). It is possible that the restoration of mutant PTEN levels might stabilize and activate WT-P53, which facilitates KML001-induced apoptosis. 

ATO has been used with other agents as a combinational therapy to increase anti-tumor effects. For example, a combination of ATO with the natural anti-cancer agent gossypol synergistically targeted glioma stem-like cells through Hedgehog and Notch signaling [34]. KML001 has been combined with doxercalciferol and gemcitabine for synergistic anti-cancer effect in acute lymphoid leukemia and pancreatic cancer cells, respectively [35,41]. KML001 has also been reported to sensitize glioblastoma when combined with temozolomide or radiation by causing increased DNA damage and apoptosis [18], although the mechanisms by which KML001 exerts its synergistic effect have not been explored. In our study, we demonstrated that KML001 alone is enough to reduce the growth of glioblastoma in vitro and in vivo, probably through Akt downregulation. However, we cannot rule out other mechanisms involved in KML001-induced cell death. Reactive oxygen species (ROS) are involved in apoptosis induced by ATO [42] and KML001 induced autophagic cell death in prostate cancer cells via ROS [43]. ROS were increased by KML001 in U87-MG cells (Supplementary Figure S9A) but N-acetyl cysteine (NAC), an antioxidant, did not reduce KML001-induced cell death (Supplementary Figure S9B). NAC did not affect the signaling pathways induced by KML001, such as downregulation and inactivation of Akt or PTEN upregulation (Supplementary Figure S9C). Signal transducer and activator of transcription 3 (STAT3) is highly expressed and overactivated in many tumors, including glioblastoma [44]; thus, targeting STAT3 could be a promising strategy for glioblastoma therapy [45]. ATO is known to inactivate STAT3 in gastric cancer cells through SHP-1 induction [46]. KML001 treatment also decreased STAT3 activation, which was not reversed by NAC treatment (Supplementary Figure S9C). How KML001 inactivates STAT3 remains to be elucidated. 

To evaluate the anti-tumor effects of KML001, seven days after tumor injection, U87-MG orthotopic xenograft mice were treated with 2 mg/kg or 5 mg/kg KML001 every other day for 14 days. KML001 treatment resulted in a significant reduction of tumor growth with decreased Akt activation (Figure 7B,D). The doses of KML001 used in our study have been reported not to be toxic in vivo when treatment is carried out every day for 20 days, as determined by body weight change and levels of AST and ALT (K-9). We also observed little body weight reduction when treated with KML001 at a two-day interval for 14 days (data not shown). These data indicate that KML001 has anti-tumor effect on glioma in mouse xenograft model, too. In conclusion, our data support the notion that KML001 has therapeutic potential for the treatment of glioma, especially when Akt is overactivated by either PTEN deletion or mutation. 

## 4. Materials and Methods

### 4.1. Cell Culture 

Human glioblastoma cell lines, U87-MG, LN229 and human glioma cell line, U251, human prostate cancer cell lines, Du145 and PC3, human breast cancer cell lines, MDA-MB231 and BT-549 were obtained from the American Type Culture Collection (Rockville, MD, USA). Roswell Park Memorial Institute (RPMI), Dulbecco’s modified Eagles medium (DMEM), Fetal bovine serum (FBS), antibiotic-antimycotic (100X) were purchased from Thermo Fisher Scientific (Waltham, MA, USA). U87-MG, U251, and LN229 cell lines were cultured in DMEM, supplemented with 10% FBS and antibiotic-antimycotic (1X). Du145, PC3, MDA-MB231, and BT-549 were cultured in RPMI, supplemented with 10% FBS and antibiotic-antimycotic (1X). 

### 4.2. Antibodies and Reagents 

KML001 was a kind gift from Komipharm International (Seoul, Korea). Anti-P53, Horseradish Peroxidase (HRP)-conjugated goat anti-mouse IgG and goat anti-rabbit IgG were purchased from Santa Cruz Biotechnology (Santa Cruz, CA, USA). Anti-phospho-Akt (Ser473), anti-Akt, anti-phospho-GSK3β, anti-PTEN, anti-caspase-3, anti-PARP, anti-β-actin were from cell signaling technology (Beverly, MA, USA). FITC annexin V apoptosis detection kit was obtained from BD Pharmingen (San Jose, CA, USA). JC-1 assay kit from Molecular Probes (Eugene, OR, USA). Immobilion-P polyvinylidene difluoride (PVDF) membranes (0.45 µm) were from Millipore (Bedford, MA, USA). Micro-BCA protein assay reagents and Chemiluminescent reagents were from Pierce (Rockford, IL, USA). MG132, Pan-caspase inhibitor, Puromycin, Akt inhibitor II were obtained from Calbiochem (La Jolla, CA, USA). Chloroquine was purchased from Sigma-Aldrich. LY294002 was purchased from LC Laboratories (Woburn, MA, USA).

### 4.3. Drug Treatment

Cells were grown to approximately 70% confluence and then serum-starved in RPMI containing 0.1% bovine serum albumin (BSA) prior to treatment. Cells were treated with the indicated concentrations of reagents in the same media.

### 4.4. Cell Viability and Cell Proliferation Assay

For the cell proliferation assay, the cells were plated in 96-well cell culture plate (5 × 10^3^ cells) and cultured overnight prior to treatment with KML001. The effects of the treatments on cell growth were determined with CellTiter 96 aqueous nonradioactive cell proliferation assay kit (MTS; 3-(4,5-dimethylthiazol-2-yl)-5-(3-carboxyme-thoxyphenyl)-2-(4-sulfophenyl)-2H-tetrazolium, Promega, Madison, WI, USA) as described in the manufacturer’s instruction. Absorbance was measured at 490 nm with a PowerWave HT Spectrophotometer (Biotek instruments, Winooski, VT, USA). For trypan blue exclusion assay, cells were plated in 6-well plates (1.5 × 10^5^ cells/well) for 24 h, followed by KML001 treatment for 24 h. The cells were trypsinized, stained with trypan blue, and counted under the microscope as viable cells. Alternatively, after staining with trypan blue, cells were counted by countess^TM^ automated cell counter from Invitrogen (Carlsbad, CA, USA). Each experiment was performed in triplicate. 

### 4.5. Immunoblotting Analysis

After washing with ice-cold phosphate-buffered saline (PBS; 10 mM Na_2_HPO_4_, pH 7.4, 145 mM NaCl, and 2.7 mM KCl), cells were lysed with 2X SDS-PAGE sample buffer (20 mM Tris, pH 8.0, 2% SDS, 2 mM DTT, 1 mM Na_3_VO_4_, 2 mM EDTA, 20% glycerol) and boiled for 5 min. Protein concentration of each sample was determined using a Micro-BCA protein assay reagent as described by the manufacturer. In all, 30 ug of total cellular protein was separated by 8%–12% SDS-PAGE and then transferred to PVDF membranes. The membranes were blocked 1 hr at room temperature in TBST (20 mM Tris, pH 8.0, 150 mM NaCl, and 0.05% Tween 20) containing either 5% BSA (for immunoblotting with anti-phospho-Akt antibody) or 5% non-fat dried milk (for immunoblotting with other antibodies). The membranes were then incubated with the primary antibody overnight at 4 °C, washed three times with TBST, incubated with HRP-conjugated goat anti-mouse IgG or goat anti-rabbit IgG secondary antibodies for 1 h at room temperature, and then washed with TBST three times. The labeled proteins were visualized using the enhanced chemi-luminescence method. In the instances in which the same membrane was reprobed with a different primary antibody, the membrane was incubated in a stripping buffer (62.5 mM Tris, pH6.8, 2% SDS and 0.75% β-mercaptoethanol) at 37 °C for 15 min, washed extensively, reblocked with 5% non-fat milk, and then reprobed with another antibody as described above. 

### 4.6. Flow Cytometer Analysis

Cells were trypsinized and suspended in PBS containing 2.5 mM EDTA, 2.5 mM EGTA and 1% BSA. For the measurement of mitochondrial membrane potential changes, cells were incubated with 20 nM 2 µM JC-1 in PBS for 15–30 min at 37 °C, followed by flow cytometer analysis (FACSCalibur; Becton Dickinson Bioscience, San Jose, CA, USA). JC-1 is a lipophillic dye and it may be the most sensitive indicator for mitochondrial membrane potential [24]. At low membrane potential, JC-1 occurs as a monomer that emits green fluorescent, and at higher membrane potential JC-1 forms aggregates that emit red fluorescence. The data were analyzed with Cell Quest Software (BD bioscience, San Jose, CA, USA).

### 4.7. Annexin V/PI Staining

For detection of apoptotic cells, cells were harvested and incubated with fluorescein isothiocyanate (FITC)-conjugated annexin V reagent and propidium iodide (PI) according to the manufacturer’s instructions for the FITC annexin V apoptosis detection kit I (BD Biosciences, San Jose, CA, USA). Cells were then analyzed using flow cytometry. The data were analyzed with Cell Quest Software (BD Biosciences).

### 4.8. Reverse Transcription—Polymerase Chain Reaction

Cellular RNA was extracted from glioblastoma cell lines using the Trizol reagent (Invitrogen, Carlsbad, CA, USA), according to the manufacterer’s instructions. cDNA fragments were amplified with the following primer pairs: AKT 5′- ATCCGCTGCCTGCAGTGGACC -3′ (sense), 5′- TTGTCCAGCATGAGGTTCTCC-3′ (antisense) PTEN 5′-ACCGCCAAATTTAATTGCAG-3′(sense), 5′-GGGTCCTGAATTGGAGGAAT-3′ (anti-sense). β-actin 5′-ACAACGGCTCCGGCATGTGCAA-3′ (sense) 5′-CGGTTGGCCTTGGGGTTCAG-3′ (antisense). The condition of PCR was as follows: (AKT, EGFR) 94 °C for 30 s, 55 °C for 30 s, 72 °C for 1 min, and (PTEN) 94 °C for 30 s, 58 °C for 30 s, 72 °C for 1 min for 30 cycles. Products were analyzed on a 1% agarose gel. 

### 4.9. Establishment of the Stable Cell Lines Overexpressing Wild Type-Akt and Dominant-Active Akt 

Constructs for wild type-*AKT*/pCMV5 and dominant active-*AKT*/pCMV5 were a kind gift from Dr. Jong-Sun Park (Chung-nam national university, Daejeon, Korea). Dominant active Akt is a membrane-targeted form of wild type-Akt as described before [47]. pLL3.7 vector was obtained from Addgene Inc. (Cambridge, MA, USA). Its U6 promoter was digested with restriction enzymes *Xba*I/*Hpa*I and then the corresponding sites were replaced by EF1α promoter/enhancer as described by Invitrogen (San Diego, CA, USA) to over-express a gene of interest. The XbaI/BglII fragment digested from either wt-*AKT*/pCMV5 or active-*AKT*/pCMV5 vector was inserted into pLL3.7 vector. The resultant wt-*AKT*/pLL3.7 or active-*AKT*/pLL3.7 constructs also co-expressed enhanced green fluorescent protein (EGFP) as a reporter gene. Recombinant lentiviruses were produced according to the manufacturer’s protocol (Invitrogen). In brief, we co-transfected pLL3.7 (for mock, wt-Akt, and active-Akt) vector and viral packing mix (Invitrogen) into 293FT cells and harvested the resulting supernatant after 48 h. Media containing live lentiviruses were collected, spun to remove cell debris, filtered through a Millex-HV syringe filter (0.45 μm, Millipore, Bedford, MA, USA), and stored at −80°C before used. The glioblastoma cells, U87-MG, LN229, cells were infected with each lentivirus supernatant and 2 μg/mL polybrene (hexadimethrine bromide). After 24 h, media was changed and then cells were incubated for 48 h before the aspiration of media. The GFP expression after cell sorting was greater than 80–90%.

### 4.10. Establishment of the Stable Cell Lines Expressing PTEN

Based on the gene sequences of *PTEN* (GenBank AF067844), the primer sequences were designed according to procedure of the pLenti6/V5-D-TOPO^®^ Cloning Kit (Invitrogen, Carlsbad, CA, USA): Forward primer: 5′-CACCATGACA GCCATCATCAAA-3′, which contains a Kozak translation initiation sequence (underlined) with an ATG initiation codon (italicized), Reverse primer: 5′- GACTTTTGTAATTTGTGTATG-3′ (synthesized by Bioneer Corporation, Daejeon, Korea). The PCR was carried out using an AccuPower TLA PCR PreMix (Bioneer Corporation, Daejeon, Korea) and *PTEN*/pCDNA3 as a template, which was kindly provided by Dr. Jong Bae, Park (National Cancer Center, Goyang, Korea). The condition of PCR was as follows: 30 cycles of 94 ℃ for 30 s, 54 ℃ for 30 s, and 72 ℃ for 1 min. The gel-purified PCR products were ligated into the pLenti6/V5-D-TOPO^®^ expression vector, resulting in the plasmid of pLenti6/V5-*PTEN*. The *PTEN* cDNA cloned in pLenti6/V5-D-TOPO vector was confirmed by restriction enzyme digestion and by DNA sequencing analysis (Macrogen, Seuoul, Korea). Lentivirus production was done with ViraPower Lentivirus Expression Systems (Invitrogen, Carlsbad, CA, USA) according to the manufacturer’s instructions. In brief, the ViraPower™ Packaging Mix and pLenti6/V5-*PTEN* were cotransfected into 293FT cells (6 × 10^6^ total cells), using Lipofectamine™ 2000 (Invitrogen, Carlsbad, CA, USA), and harvested the resulting supernatant after 72 h. The glioblastoma cells, U87-MG was infected with equivalent titers of control, *PTEN* viruses and were incubated for at least 48 h. Stable cell lines of *PTEN* were selected with fresh media containing blasticidin.

### 4.11. Establishment of Orthotropic Xenograft 

U87-MG cells were orthotopically transplanted following washing and re-suspension in PBS (1 × 10^5^ cells per mouse). Cells were injected stereotactically into the left caudate-putamen region of 6-week-old female Balb/c nude mice (n = 5 in each group) as described before [48]. The injection coordinates were 2.2 mm to the left of the midline and 0.2 mm posterior to the bregma at a depth of 3.5 mm. After 1-week injection, mice were treated either without (control) or with KML001 for 2 weeks (two-day intervals) orally. After 2 weeks of treatment the brain of each mouse was harvested and fixed in 4% paraformaldehyde. All animal research was conducted in accordance with protocols approved by the Institutional Animal Care and Use Committee at the National Cancer Center, Republic of Korea (NCC-10-005D, approved on 2 March 2010).

### 4.12. In Vivo Imaging and Analysis In Vivo MR Imaging

Mice were imaged with a 7.0-T MR imaging system (BioSpec 70/20 USR; Bruker Biospin, Ettlingen, Germany). Mice were anesthetized with 2% isoflurane gas, and their respiration rate was monitored during examinations. Anatomic images were acquired by using a rapid acquisition with relaxation enhancement (RARE) sequence and the following parameters: repetition time msec/echo time msec, 2600/30; number of averages, two; RARE factor, four; matrix, 256 × 256; slice thickness, 0.7 mm; slice distance, 1 mm; and field of view, 2.0 × 2.0 cm. the arbitrary shape region of interest (ROI) spanning a whole tumor region were drawn on the MR images. The tumor volumes were calculated by multiplying the area of the ROI’s and the slice distance (mm^3^).

### 4.13. Immunohistochemical Staining

The brains were fixed with 4% paraformaldehyde for 24 h at 4 °C. For immunostaining, after the antigen retrieval process with citrate buffer (pH 6.0) and endogenous peroxidase blocking with 3% hydrogen peroxide, tissue sections were incubated in 1% BSA blocking solution (*v*/*v*) for 30 min at room temperature, then in primary antibody overnight at 4 °C in a humidified chamber. For primary antibodies, we used rabbit antibody to pAKT (Cell Signaling Technology, 1:500). Sections were rinsed three times with a washing buffer (1% BSA, 0.1% cold fish skin gelatin, 0.5% Triton X-100, and 0.01 M PBS) and then incubated biotinylated secondary antibody (diluted 1:200, Vector Laboratories; Burlingame, USA) overnight at 4 °C. An avidin–biotin peroxidase enzyme complex was prepared and applied according to manufacturer’s instructions (Vectastain Elite ABC kit). Finally, sections were incubated for 5 min in a DAB/hydrogen peroxide substrate solution (prepared according to manufacturer’s instructions, Vectastain DAB substrate kit, Vector Laboratories). Sections were mounted in an aqueous mountant (Vectashield, Vector Laboratories) and observed under a microscope (Olympus, Tokyo, Japan). For quantification of IHC staining, three-staining fields were selected randomly from each section. The average IOD (Integrated Optical Density) levels between treated groups were then compared. Image-J software was used for analysis. 

### 4.14. Immunofluorescence Analysis

Cells were fixed with 1% paraformaldehyde in PBS at 4 °C for 20 min, permeabilized and blocked in 3% BSA in PBS for 1 h at room temperature. Cells were then incubated with anti-p53 antibody for 4 °C overnight, and then incubated with Alexa 488-conjugated antibody for 1 h at room temperature. For nucleus staining, cells were incubated with DAPI in PBS. Cells were examined using confocal microscopy (Zeiss, Jena, Germany).

### 4.15. Statistical Analysis

Comparison between two groups were performed using Student’s *t*-test. Multiple group comparisons were made parametric one-way ANOVA followed post hoc test (Bonferroni correction). Data represent average values and standard deviations (error bars) obtained from three independent experiments.

## 5. Conclusions

Glioblastoma is frequently associated with PTEN loss or mutation, which leads to AKT activation. KML001, sodium arsenite induced growth inhibition due to apoptosis of glioma cell lines more sensitively in PTEN-deficient cells with high-AKT activity than in PTEN-wild type cells. KML001 inactivates AKT via proteasomal degradation of AKT. Additionally, in vivo anti-tumor effects of KML001 was observed in orthotopically xenografted model. These data indicate that KML001 could be an effective chemotherapeutic drug depending on higher Akt activity due to PTEN deletion/mutation in glioblastoma cancer patients.

## Supplementary Materials

The following are available online at https://www.mdpi.com/1422-0067/21/23/8982/s1.
