# Supplementary material for: Anti-Tumor Effects of Sodium Meta-Arsenite in Glioblastoma Cells with Higher Akt Activities
Source: Int J Mol Sci. 2020 Nov 26;21(23):8982. doi: 10.3390/ijms21238982 (PMC7729740; doi:10.3390/ijms21238982)

Supplementary Information

Materials and Methods

Cell Culture

Human glioblastoma cell lines, U87-MG, U251, and LN229 were obtained from the American Type Culture Collection (Rockville, MD). Dulbecco's modified Eagles medium (DMEM), Fetal bovine serum (FBS), antibiotic-antimycotic (100X) were purchased from Thermo Fisher Scientific (Waltham, MA). U87-was cultured in DMEM, supplemented with 10% FBS and antibiotic-antimycotic (1X)

Antibodies and Reagents

KML001 was a kind gift from komipharm international (Seoul, Korea). Horseradish Peroxidase (HRP)-conjugated goat anti-mouse IgG and goat anti-rabbit IgG were purchased from Santa Cruz Biotechnology (Santa Cruz, CA). Anti-phospho-Akt (Ser473), anti-Akt, anti-PTEN anti-phospho- STAT3, anti-β-actin were from cell signaling technology (Beverly, MA)

Immunoblotting

After washing with ice-cold PBS, cells were lysed with 2X SDS sample buffer (20 mM Tris, pH 8.0, 2% SDS, 2 mM DTT, 1 mM Na3VO4, 2 mM EDTA, 20% glycerol) and boiled for 5 min. Protein concentration of each sample was determined using a BCA protein assay reagent (Pierce, Rockford, IL) as described by the manufacturer. In all, 20-40 μg of total cellular protein was separated by 8-12% SDS-PAGE and then transferred to PVDF membranes. The membranes were blocked for 1hr at room temperature in tris-buffered saline and tween 20 (TBS-T, 20 mM Tris, pH 8.0, 150 mM NaCl, and 0.05% Tween 20) containing 5% non-fat

dried milk. The membranes were then incubated with the primary antibody for overnight at 4℃, washed three times with TBS-T, incubated with HRP-conjugated goat anti-mouse IgG or goat anti-rabbit IgG secondary antibodies for 1hr at room temperature, and then washed three times with TBS-T. The labeled proteins were visualized using the chemi-luminescence method.

Cell viability assay

For the cell viability assay, the cells were plated in 96-well cell culture plate ($5x10^3$ cells) and cultured overnight prior to treatment with KML001. The effects of the treatments on cell growth were determined with celltiter 96 aqueous nonradioactive cell proliferation assay kit (MTS; 3-(4, 5-dimethylthiazol-2-yl)-5-(3-carboxyme-thoxyphenyl)-2-(4-sulfophenyl)-2H-tetrazolium, Promega, Madison, WI) as described in the manufacturer's instruction. Absorbance was measured at 490 nm with a powerwave HT spectrophotometer (Biotek instruments, Winooski, VT).

Immunofluorescence and Confocal Microscopy Imaging

Cells grown on cover slips were fixed with 3.7% paraformaldehyde in PBS at 4℃ for 20 min, rinsed with PBS. The cells were permeabilized and blocked in 3% BSA in PBS for 1 hr at room temperature, and then washed in PBS. Cells then stained Alexa 488- conjugated p53 antibody for 1 hr at room temperature. The cover slips were washed in PBS and mounted on glass slides. For nucleus staining, cells were incubated with DAPI in PBS. After washing with PBS, cells were examined using Carl Zeiss fluorescence microscopy or inverted laser-scanning microscopy (Carl Zeiss MicroImaging, Göttingen, Germany).

ROS measurements

U87-MGcells grown in attachment or suspension conditions were incubated with 20 mM of CM-H2DCFDA (Thermo Fisher Scientific, Waltham, MA) for 40 min at 37℃ and collected, and analyzed by flow cytometry using a FACSCalibur (Becton Dickinson Bioscience, San Jose, CA, USA).

Data analysis

All data points represented the mean value of at least three independent experiments with triplicates for each. Statistical significance was determined by Student's *t*-test with $p<0.005$.

Figure legends

Supplementary Figure 1. Effects of KML001 on cell growth in human glioblastoma cell lines. Cells were treated with 0-30 μM KML001 in the culture media for 24 hrs. Cell growth was measured by MTS assay. Values are the means ±SEM. Data are presented as mean ± SE of three independent experiments. *P<0.05, **P<0.001 and indicate significant difference from the control. Similar results were observed in three independent experiments.

Supplementary Figure 2. Densitometry analysis of immunoblots in Figure 3D The histogram shows the densitometric measuring of pAkt, Akt, pGSK3β, pro-caspase3 and PARP expression relative to β-actin. Values are the means ±SEM. Data are presented as mean ± SE of three independent experiments. *P<0.05 and indicate significant difference from the control.

Supplementary Figure 3. Effects of KML001 on HSP90 expression (A) U87-MG cells were treated with 0-30 μM KML001 for 24 hrs and cell lysates were subjected to immunoblotting analysis using the indicated antibodies. Similar results were observed in three independent experiments. The histogram shows the densitometric measuring of HSP90 and Akt expression relative to β-actin. Values are the means ±SEM. Data are presented as mean ± SE of three independent experiments. *P<0.05 and indicate significant difference from the control.

Supplementary Figure 4. Effects of lactacystin on KML001-induced Akt downregulation **(A)** U87-MG cells were treated without or with 10 μM KML001 for 24 hsr and KML001-treated cells were incubated with 5 μM or 10 μM lactacyctin for indicated times before harvest. Cell lysates were subjected to immunoblotting analysis using indicated antibodies. The histogram shows the densitometric measuring of AKT expression relative to β-actin. Values are the means ±SEM. Data are presented as mean±SE of three independent experiments.

Supplementary Figure 5. **Densitometry analysis of immunoblots in Figure 4** The histogram shows the densitometric measuring of Akt expression relative to β-actin (Figure 4C). Akt expression relative to β-actin (Figure 4D). PTEN expression relative to β-actin (Figure 4E and 4F). Values are the means ±SEM. Data are presented as mean ± SE of three independent experiments. *P<0.05 and indicate significant difference from the control

Supplementary Figure 6. Densitometry analysis of immunoblots in Figure 5 The histogram shows the densitometric measuring of PTEN, pAkt (S473), pAkt (T308) and Akt expression relative to β-actin (Figure 5A). The histogram shows the densitometric measuring of pAkt and Akt expression relative to β-actin (Figure 5D). Values are the means ±SEM. Data are presented

as mean ± SE of three independent experiments. *P<0.05 and indicate significant difference from the control.

Supplementary Figure 7. Densitometry analysis of immunoblots in Figure 6A and 6C The histogram shows the densitometric measuring of pAkt, Akt and PTEN, expression relative to β-actin (Figure 6A). The histogram shows the densitometric measuring of cleaved PARP, pAkt, Akt and PTEN expression relative to β-actin (Figure 6D). Values are the means ±SEM. Data are presented as mean ± SE of three independent experiments. *P<0.05 and indicate significant difference from the control.

Supplementary Figure 8. Effects of KML001 on p53 expression (A) U87-MG cells were treated with 0-30 μM KML001 for 24 hrs and cell lysates were subjected to immunoblotting analysis using the indicated antibodies. Similar results were observed in three independent experiments. The histogram shows the densitometric measuring of p53, p-p53, PUMA and Bax expression relative to β-actin. Values are the means ±SEM. Data are presented as mean ± SE of three independent experiments. *P<0.05 and indicate significant difference from the control. (B) U87-MG cells were treated with treated with 10 or 20 μM KML001 for 24 hrs and stained with anti-p53 antibody and DAPI and then analyzed by confocal microscopy analysis. Isotype IgG was used as a control. Magnification: x 400, Scale bars = 20 μm.

Supplementary Figure 9. Effects of ROS induced by KML001 treatment on cell growth inhibition and cell signalings. (A) U87-MG cells were treated with treated with 5, 10 or 20 μM KML001 for 24 hrs and ROS levels were measured by flow cytometry using a ROS-sensitive dye, CM-H2DCFDA. (B) U87-MG cells were treated either with 10 μM KML001 or with either 1-10 mM of NAC, a ROS scavenger for 24 hrs and cell numbers were counted by trypan

blue exclusion assay. (C) U87-MG cells were treated either with 10 μM KML001 or with either 1-10 mM of NAC, a ROS scavenger for 24 hrs and cell lysates were subjected to immunoblotting analysis using the indicated antibodies. Similar results were observed in three independent experiments. The histogram shows the densitometric measuring of pAkt, Akt, PTEN and pSTAT3 expression relative to β-actin. Values are the means ±SEM. Data are presented as mean ± SE of three independent experiments. *$P<0.05$ and indicate significant difference from the control.

Supplementary Figure. 1

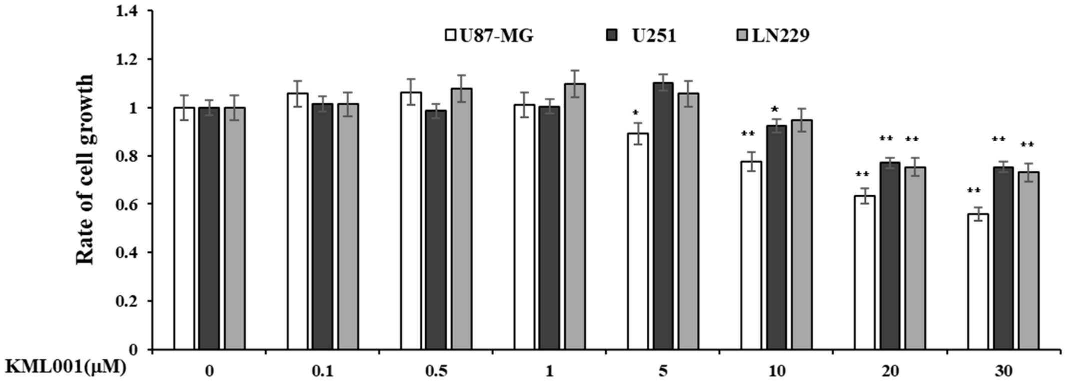

Supplementary Figure. 2

Figure 3D

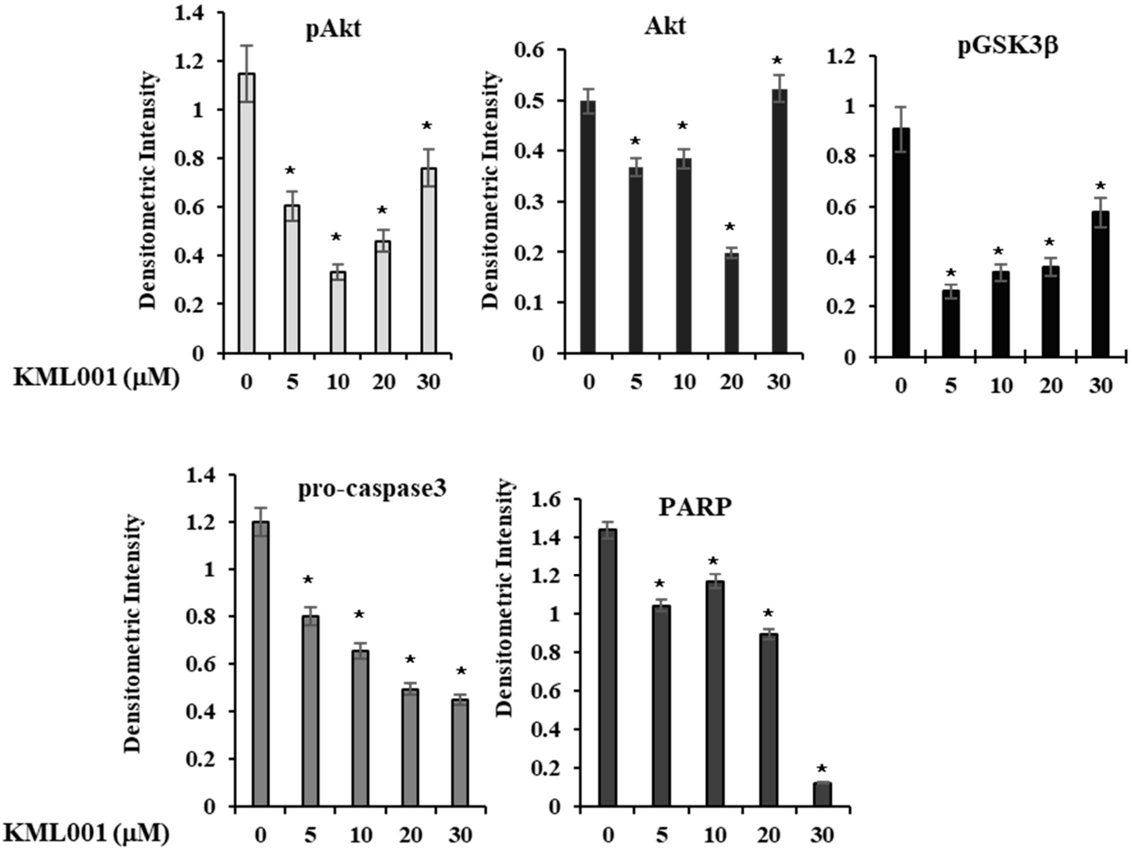

Supplementary Figure. 3

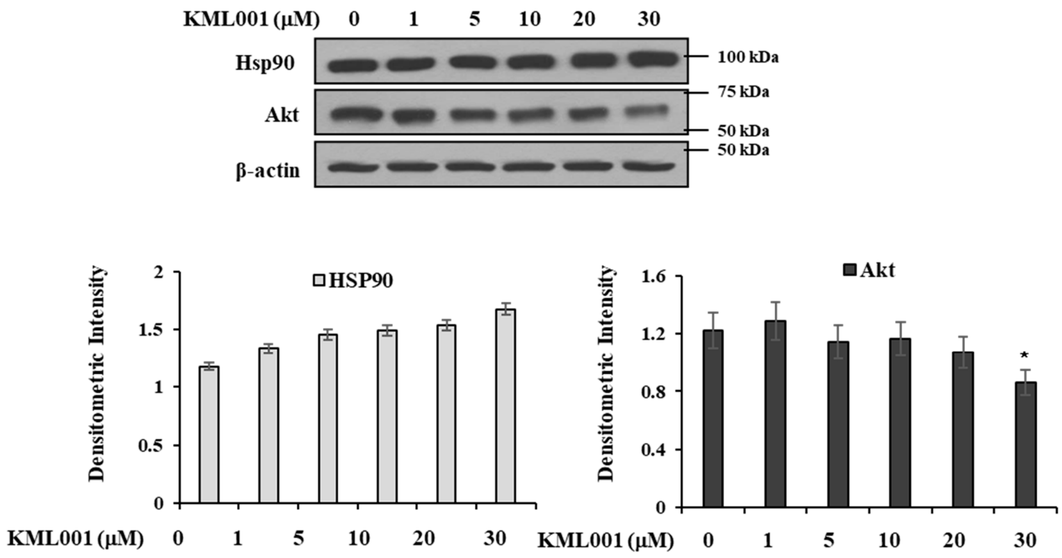

Supplementary Figure. 4

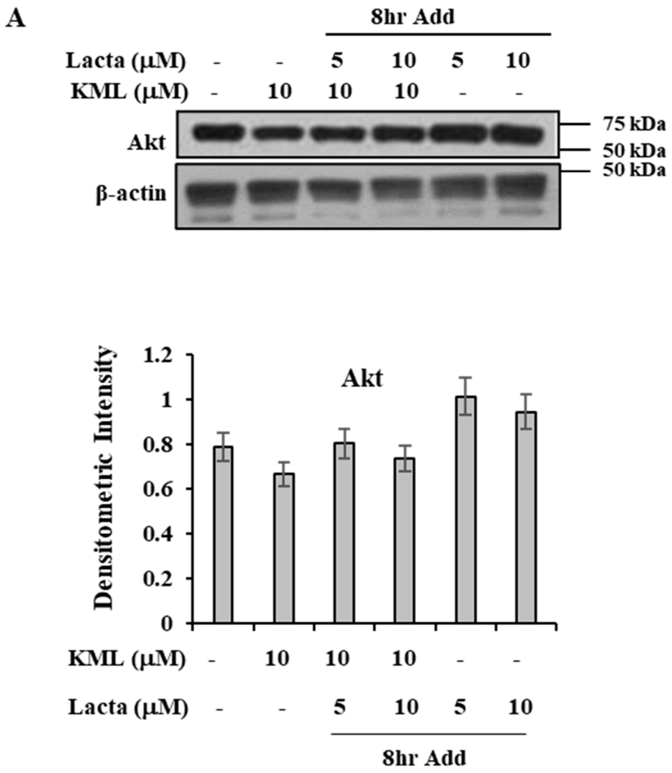

Supplementary Figure. 5

Figure 4C

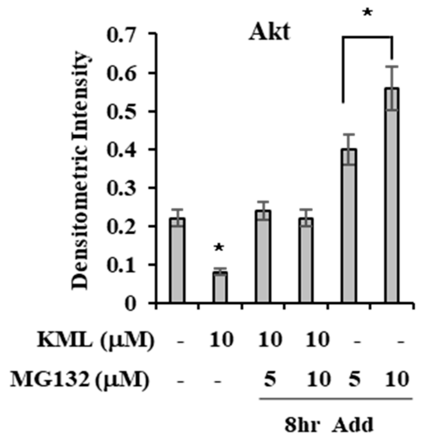

Figure 4E

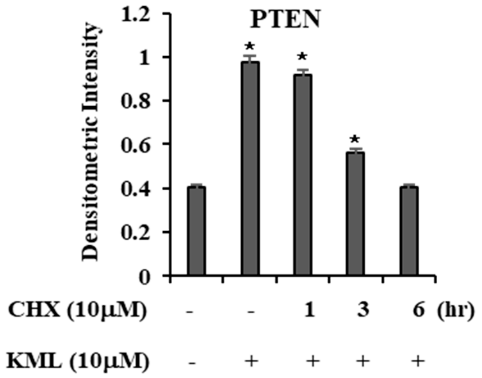

Figure 4D

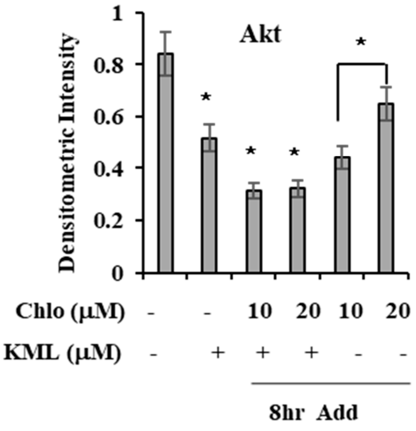

Figure 4F

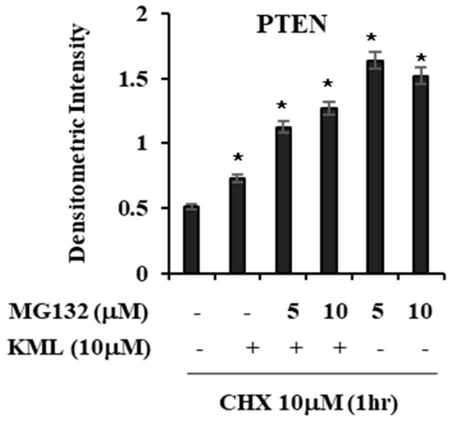

Supplementary Figure. 6

Figure 5A

U87-MG

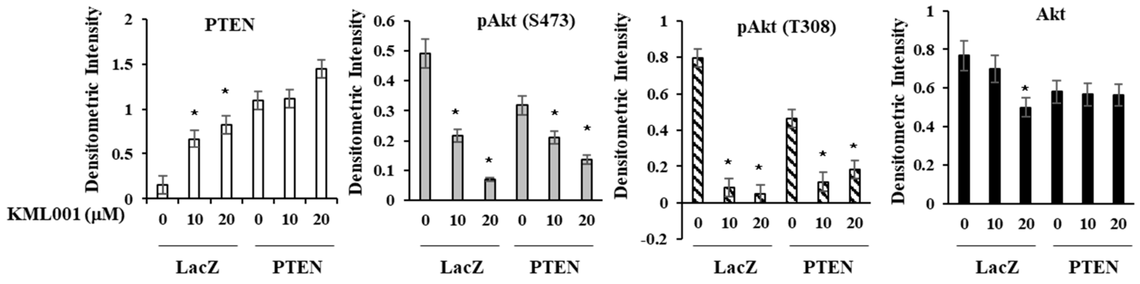

Figure 5D

LN229

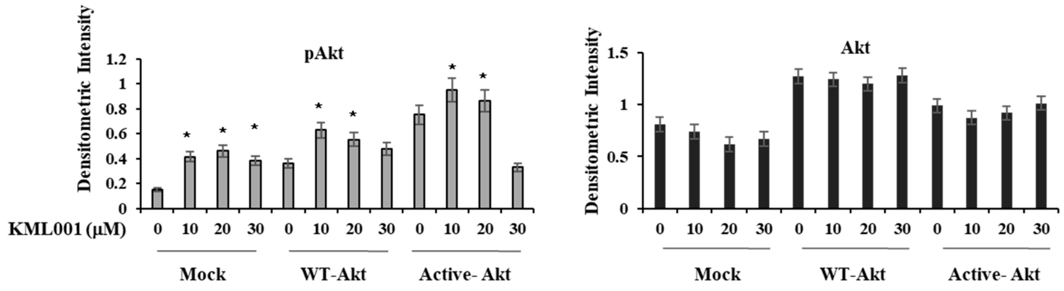

Supplementary Figure. 7

Figure 6A

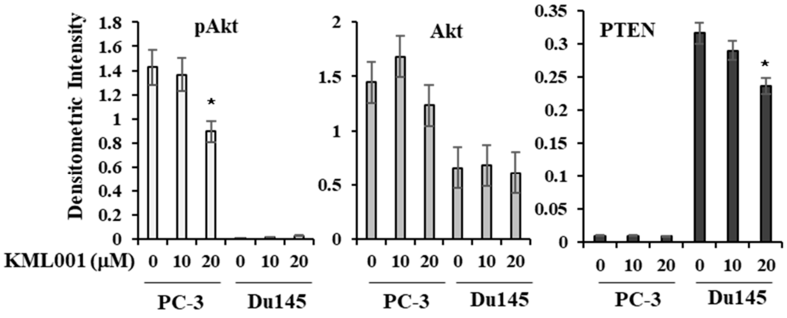

Figure 6C

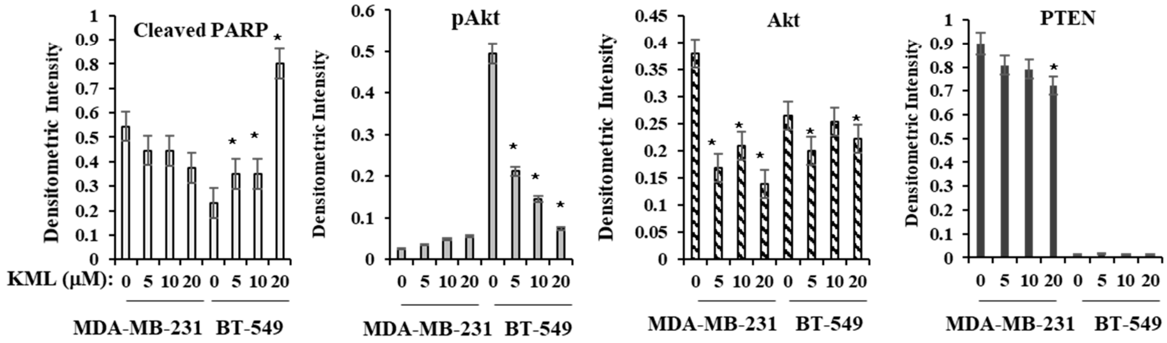

Supplementary Figure. 8

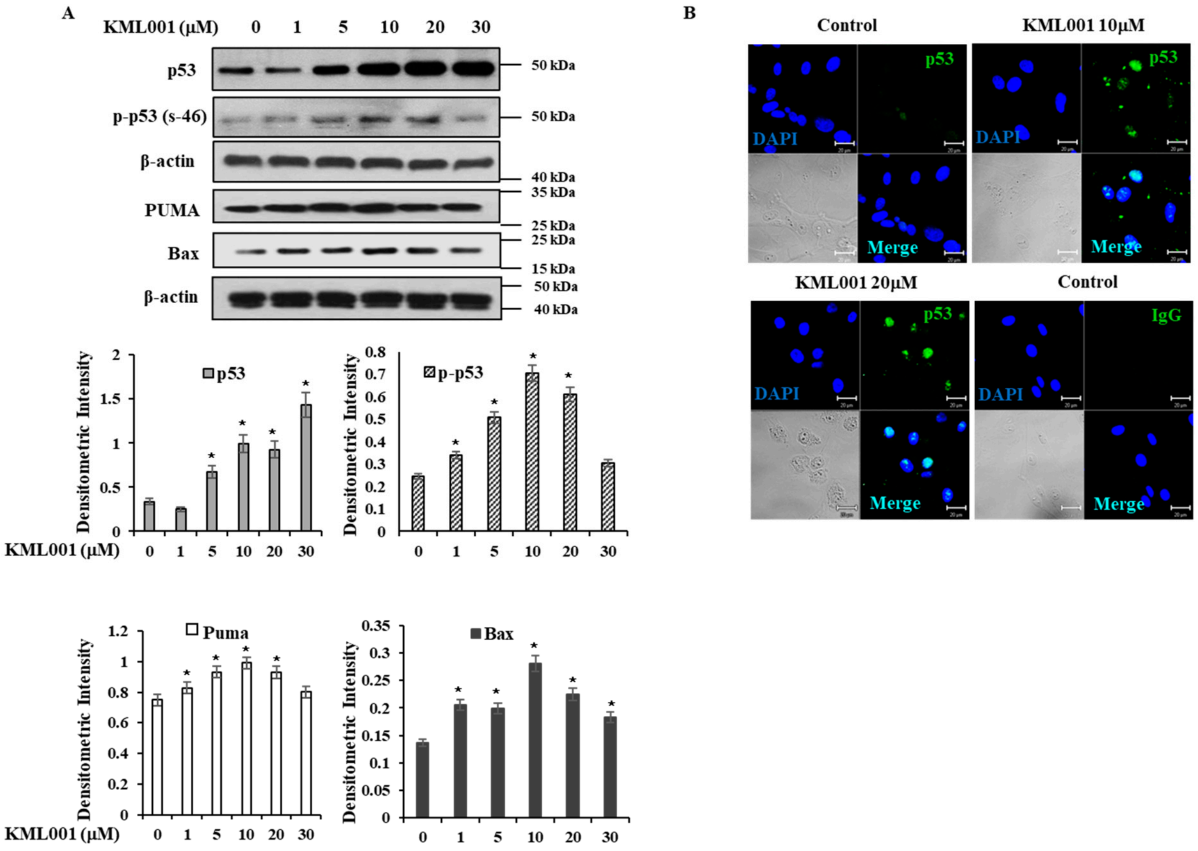

Supplementary Figure. 9

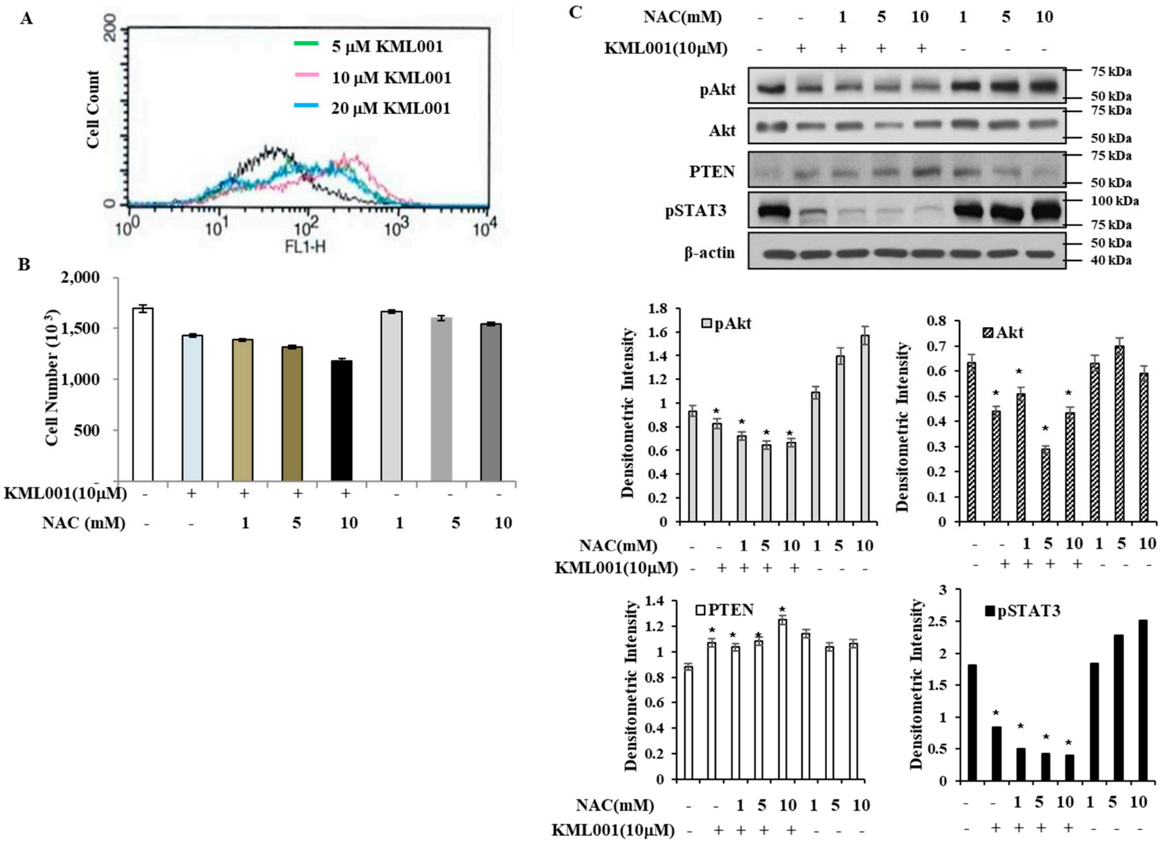

Supplement: Supplementary file 1 [file ijms-21-08982-s001.pdf]
